# Recent Developments of Nano Flame Retardants for Unsaturated Polyester Resin

**DOI:** 10.3390/ma17040852

**Published:** 2024-02-11

**Authors:** Adriana Dowbysz, Mariola Samsonowicz, Bożena Kukfisz, Piotr Koperniak

**Affiliations:** 1 Department of Chemistry, Biology and Biotechnology, Bialystok University of Technology, Wiejska 45A Street, 15-351 Bialystok, Poland; adriana.dowbysz@pb.edu.pl; 2 Institute of Safety Engineering, Fire University, Slowackiego Street 52/54, 01-629 Warsaw, Poland; bkukfisz@apoz.edu.pl; 3 Lukasiewicz Research Network—Institute of Aviation, 110/114 Krakowska Avenue, 02-256 Warsaw, Poland; piotr.koperniak@ilot.lukasiewicz.gov.pl

**Keywords:** flame retardant, nanoparticle, nanocomposite, thermal degradation, flammability

## Abstract

For many years, efforts have been made to reduce the flammability of unsaturated polyester resins (UPRs), which are often used in the rail, shipbuilding, and construction industries. Without modification, they often fail to meet fire safety standards. Despite a rich history of flame retardants (FRs) applied to UPRs, researchers seek new solutions that will provide lower flammability and smoke density, as well as attaining a lower environmental impact from the composites. The objective of the study is to highlight the most important recent research on promising nano FRs in order to promote their further development. Mechanisms of action of several groups of nano FRs, such as clay-based, carbon-based, transition metal compounds, layered double hydroxides, polyhedral oligomeric silsesquioxanes, and others, including bio-based, have been studied. Particular emphasis has been laid on nano FRs applied to UPRs, and their influences on thermal stability, flammability, and mechanical properties. Moreover, the environmental impact and toxicity of nano FRs have been discussed. Results have proved that nano FRs applied at low loadings may significantly improve thermal stability, with a simultaneous increase or only a slight decrease in mechanical properties. However, attention on related environmental issues has highlighted the necessity of carefully selecting novel nano FRs.

## 1. Introduction

Unsaturated polyester resins (UPRs) are widely applied thermoset resins whose structure contains linear polycondensation products. Despite numerous advantages, such as low price, chemical, water, and aging resistance, and low shrinkage [1], their main disadvantage is poor thermal resistance. Their thermal degradation starts at ca. 350 °C, which is accompanied by the release of large amounts of smoke. They pose a serious fire hazard and therefore are widely modified with flame retardants (FRs). Among various groups of FRs, such as inorganic hydroxides, tin and zinc compounds, or phosphorus- and nitrogen-containing compounds—which have an adverse effect on processability and mechanical properties when applied at high loadings [2]—nano FRs have received scientists’ attention. Application at low loadings may significantly improve their thermal properties and decrease flammability, with a negligible influence on the mechanical properties. Therefore, further development of UPR materials with enhanced flame-retardant properties and preserved or enhanced mechanical properties can be ensured precisely through the use of nano flame retardants [3].

Recently, nanotechnology has been an area of interest for many researchers. Due to the various properties of nanoparticles, their ability to significantly influence the properties of materials at low loadings, and their low-toxicity or nontoxic impact on human health and the environment, nanoparticles are a subject of ongoing research in different fields of study. The use of nanoparticles in fire protection is of great interest at present. Nanotechnology is applied in direct fire protection (e.g., personal protective equipment of firefighters, respiratory systems) and indirect fire protection, which refers to the design of buildings and infrastructure that meet fire safety standards [4].

**Figure 1:**
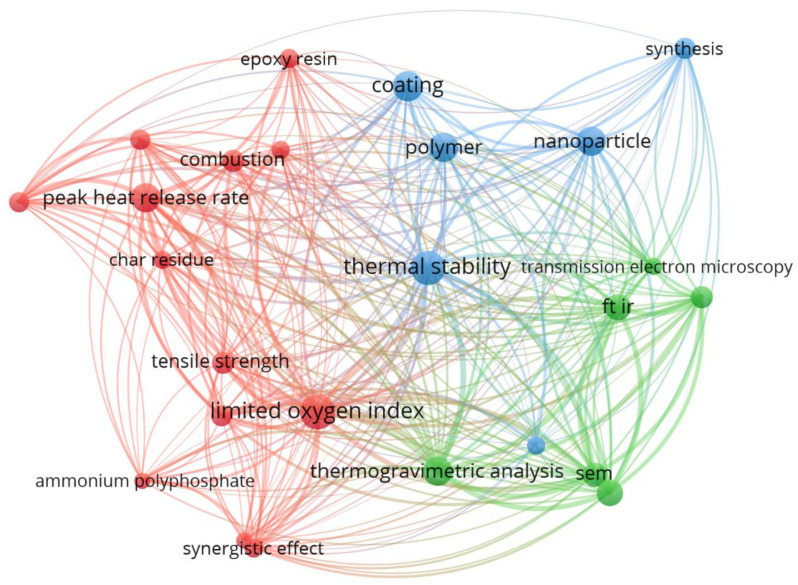
Co-occurrence of selected keywords in articles in 2010–2022 (search terms: “flame retardant” and “nano”); created with VOSviewer version 1.6.20.

A visualization of the bibliographic network based on data from the Scopus citation library in terms of the co-occurrence of selected keywords is presented in Figure 1. Circles and label sizes visualize the weights of co-occurrences of the keywords “flame retardant” and “nano”.

The figure shows that the most attention is paid to the thermal stability of nano FRs measured in terms of thermal stability by selected methods including the thermogravimetric analysis combined with the Fourier transform infrared spectroscopy, limited oxygen index (LOI), and cone calorimetry. Further research includes transmission electron microscopy and scanning electron microscopy, as well as mechanical properties testing. Nano FRs research mostly revolves around polymers, with a special emphasis on epoxy resins and their application to coatings.

The purpose of this review is to present the most recent developments, as well as summarize current knowledge on the applicability of nanoparticles as FRs for UPR. Moreover, the environmental impact of nano FRs is discussed. Promising directions for future research are indicated, with the identification of possible limitations.

## 2. Nanoparticle Classification, Synthesis Methods, and Application

**Figure 2:**
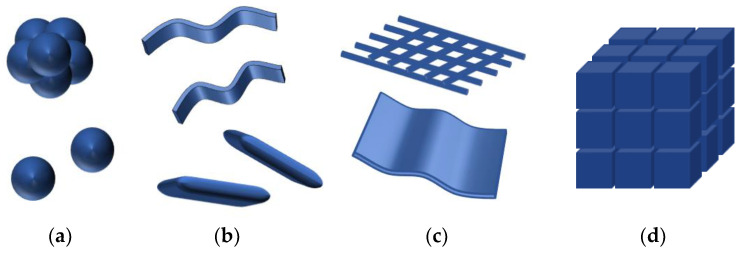
Nanoparticles classification according to the number of dimensions: (**a**) 0D spheres and clusters; (**b**) 1D fibers, wires, and rods; (**c**) 2D plates and networks; (**d**) 3D nanomaterials [5].

Nanoparticles may be divided according to several criteria, including size and number of dimensions, origin, structure, pore diameter, and potential toxicity. The former is probably the most common, and its subdivision is presented in Figure 2.

All three dimensions of 0D nanomaterials do not exceed 100 nm; additionally, they do not exhibit dimensions higher than 10 nm. Two of the three dimensions of 1D nanomaterials are in the nanoscale range, and the third is greater than 10 nm. The 2D nanomaterials have only one dimension in the nanoscale. Although all three dimensions of 3D nanomaterials are greater than 100 nm, blocks that build the structure are on a nanometer scale (from 1 to 100 nm) [6].

**Figure 3:**
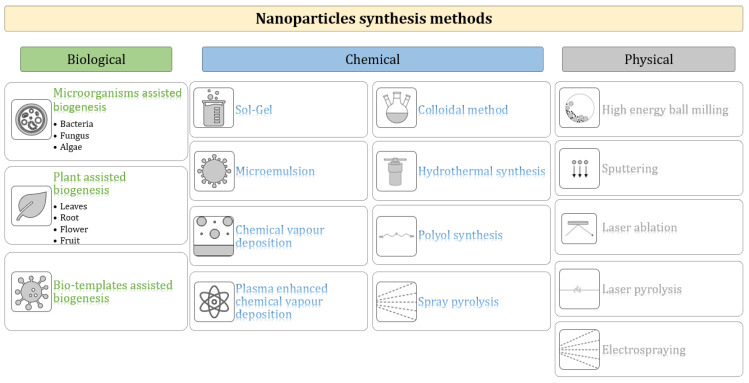
Nanoparticles synthesis methods [12,13,14,15].

Nanoparticles can be synthesized via top–down or bottom–up approaches. The top–down approach is destructive and involves dividing the bulk materials to form nanomaterials. This approach is adopted in methods such as ball milling, laser ablation, sputtering [7], and electrospraying [8]. The bottom–up approach is constructive and involves the building of nanomaterials from atoms [6]. This approach is adopted in methods such as sol-gel synthesis, chemical vapor deposition, laser pyrolysis [7], hydrothermal synthesis, biogenesis [6], microemulsion [9], spray pyrolysis [8], colloidal synthesis [10], and the polyol method [11]. The classification of nanomaterials synthesis methods into biological, chemical, and physical categories is presented in Figure 3.

The individual and unique characteristics of nanoparticles, including their considerable surface area, great mechanical strength, optical activity, and chemical reactivity, enable their usage for various purposes. In medicine and pharmaceuticals, nanoparticles may deliver the optimum amounts of drugs, increasing their therapeutic efficiency and decreasing side effects. They may also enhance MRI contrast and participate in tissue repair or the detoxification of biological fluids. Nanoparticles are also applied in different industries, such as the microelectronics, aerospace, food processing, packaging, energy, and mechanical industries, depending on their physicochemical properties [16].

Nanofillers can affect the properties of materials, allowing them to be used for specific applications with more stringent requirements. For UPR, e.g., graphene oxide and its derivatives significantly improve the fracture energy, answering the problem of brittleness [17]; zinc sulfide enhances electric and magnetic properties, through the enhancement of the dielectric constant [18]; iron (II)oxide, titanium (II)oxide, and nickel ferrite enhance the optical and mechanical properties of UPR composites [19].

Among the constantly growing areas in which nanoparticles are being used is fire safety, due to their thermal stability and their ability to form a protective char layer on the surface of polymers or composites, which reduces the heat and mass exchange. Composites based on pure UPR often do not meet the criteria specified in the construction, shipbuilding, or rail industries; the enhancement of flame-retardant properties is therefore crucial if we are to expand their applications.

## 3. Flame-Retardant Mechanisms of Nanoparticles

The improvement of flame retardancy of composites may be achieved via different routes. FRs may be blended with a polymer matrix (additive), where they may react with the polymer and build up into its structure (reactive) [14], or they may be a part of the intumescent system coated on the surface. Another way in which FRs can be incorporated is through chemically modifying the structure of the fibers or incorporating them into their structure [15].

**Figure 4:**
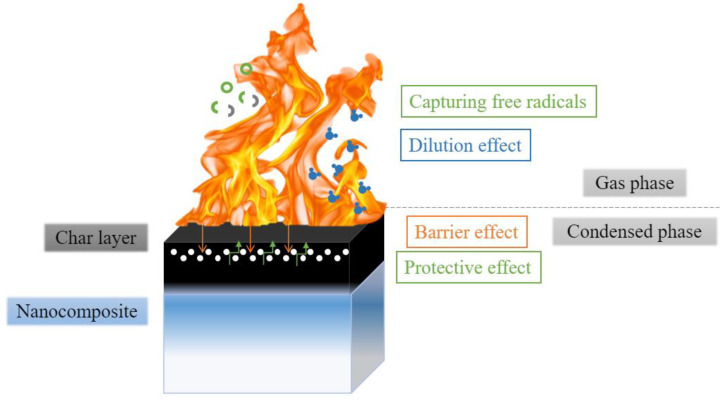
Flame-retardancy mechanisms of nanomaterials.

Nanoparticles reduce the flammability of composites via several mechanisms, including char formation and barrier effect, capturing radicals in the gas phase, or release of non-combustible volatiles diluting the concentration of combustible gases [20,21]. All the mechanisms are presented in Figure 4.

The char formed on the surface of the composite stands as a physical and diffusion barrier [21], and acts like a thermal insulation [20]. It reduces the heat flux that reaches the composite surface and reduces the gas flow from the composite to the flame.

The barrier effect, which occurs especially in nanoclays, also influences the diffusion processes. Due to the formation of a tortuous path, degraded molecules diffuse from composite to flame more slowly, and the thermal insulation of the nanocomposite is enhanced. The dispersion of nanoparticles is crucial in obtaining a fine barrier effect. Poor dispersion leads to the formation of a discontinuous layer, which is much less effective [21].

Capturing free radicals occurs in the gas phase; their trapping stops or slows the pyrolysis processes [20]. Moreover, these reactions may lead to an increase in melt viscosity, and thus an increase in the energy and time required for small degraded fragments to spread [21].

The endothermic processes of the degradation of some nanoparticles with the release of water molecules cool and rehydrate the nanocomposite, as well as dilute the concentration of combustible gases in the flame zone [20].

## 4. Nanoparticles as Flame Retardants for UPR

Nano FRs account for a fairly large percentage of research papers discussing FR containing composites (21.2%), and constitute the third largest group after organic (28.9%) and inorganic FRs (24.6%) based on research carried out between 1992 and 2022 [22]. The growing interest in FR nanocomposites is mostly due to the low loading of nano FR needed to achieve a proper level of fire protection, as compared to traditional FRs.

There are several groups of nano FRs, including clay-based, carbon-based, silica-based, metal oxides, layered double hydroxides, and others, such as bio-sourced nano FRs [23,24,25]; among these, the latter have not been studied in detail as additives to UPRs, but have been successfully incorporated to other thermosetting resins, such as epoxy [26,27]. Nano FRs are also widely applicable for other polymers [28], as well as for wood [29,30,31], standing as a sustainable construction material.

The nanoparticles’ form, content, and synthesis methods, and the effects of the application of the nano FRs in UPRs on thermal stability and mechanical properties have been summarized in Table S1. The combustion characteristics of mentioned in the article nano FRs with regard to pure UPR had been summarized in Table S2.

### 4.1. Clay-Based

Besides the most commonly used nanoclay in polymer composites, which is montmorillonite (MMT), there are several other classes of clay-based materials, including, e.g., halloysite and bentonite [24]. Units of nanoparticles of these mineral silicates are layered, and they form complex structures. For example, the layer of MMT consists of an octahedral sheet containing Al and Mg bonded with oxygen and a hydroxyl group, whereas the tetrahedral sheet consists of a silicon–oxygen tetrahedra bonded with octahedra [32]. Clay-based nanoparticles are often modified in order to achieve greater dispersion in the polymer matrix through the modification of the initial interlayer inorganic cations to become organic [24].

The mode of action of clay-based FRs is attributed to the barrier mechanism, due to the migration of nanoparticles to the surface of the composite. When they contain certain metals, such as Fe, in their structure, they may also capture free radicals [25]. The loading of clay-based nanoparticles usually is in a range from 0.1 to 10 wt.%.

#### 4.1.1. Ionic-Liquid-Functionalized Imogolite Nanotubes

Imogolite nanotubes consist of hydrous aluminosilicates with a general formula of (OH)_3_Al_2_O_3_SiOH. The inside of the nanotube is formed by Si-OH, and the outside is formed by Al_2_-OH. The imogolite nanotubes itself does not easily disperse in the hydrophobic UPR matrix; thus, Zhu et al. [33] introduced them into the ionic liquid and studied its synergism with ammonium polyphosphate (APP) in terms of the flame retardancy of UPRs.

The ionic-liquid-functionalized imogolite nanotubes were prepared via the two-step synthesis method. The imogolite nanotubes were prepared by the Arancibia–Miranda method with the use of tetraethoxysilane, aluminum nitrate nonahydrate, and ammonia, followed by their modification by 1-butyl-3-methylimidazolium hexafluorophosphate and 3-aminopropyltriethoxysilane according to the Wan method [34].

The addition of APP greatly improves the thermal stability of UPR. The thermal degradation of the pure UPR and the UPR modified with APP or APP and imogolite nanotubes comprises three stages of degradation. However, at the first step (from 200 °C to 400 °C), the pure UPR loses up to 70 wt.% of mass and the modified UPR loses up to 50 wt.% of mass. The second and third degradation steps occur at lower temperature ranges for pure UPR (from 450 °C to 550 °C and from 550 °C to 630 °C, respectively) compared to modified UPR (from 500 °C to 600 °C and from 650 °C to 730 °C, respectively); the latter also demonstrates significantly lower mass loss than modified UPR. Moreover, the values of the char residue at 730 °C for UPR modified with APP (28.2%) and UPR modified with APP and imogolite nanotubes (29.3%) are greatly increased compared to pure UPR (9.83%), showing their better char-forming ability.

Although the addition of APP increases the LOI value of UPR from 20.8% to 25.8%, the increase in LOI is even higher with the addition of both additives APP and imogolite nanotubes simultaneously (28.0%). Thus, the synergistic effect of APP and imogolite nanotubes is observed.

According to the cone calorimeter test results, the peak heat release rate (pHRR) value of pure UPR (666.25 kW/m^2^) has been reduced by 22.4% compared to a UPR/APP composite (516.68 kW/m^2^), and by 41.1% compared to UPR/APP/imogolite nanotubes (392.46 kW/m^2^).

#### 4.1.2. Organic-Modified Montmorillonite with Methyl Dihydroxyethyl Hydrogenated Tallow Ammonium

The fire performance of glass polyester/UPR composites containing 0–5 wt.% commercially available organo-modified montmorillonite (OMMT) with methyl dihydroxyethyl hydrogenated tallow ammonium (Sigma-Aldrich no. 682840, St. Louis, MO, USA) was investigated by Nguyen et al. [35].

The cone calorimeter study revealed that, as the OMMT content increases, the pHRR value decreases, with the greatest decrease of 34% obtained for the composite containing 5 wt.% of nanofiller. Similar effects were observed in the total heat release (THR) values. However, due to the presence of organic surfactant in the OMMT structure, the time to ignition is reduced for nanocomposites compared to nonmodified composites (32 s). The shortest time to ignition was observed for the composite modified with 5 wt.% of OMMT (23 s).

The reduction in the fire growth index (FGI), observed for the nanocomposites containing 1, 3, and 5 wt.% of nanofiller, stresses the importance of the proper dispersion of nanoparticles in the polymer matrix. The observed reduction in FGI by 38%, 48%, and 50%, respectively, shows that, for the highest loading of OMMT, the dispersion method by ultrasonication that was proposed by the authors is insufficient.

### 4.2. Carbon-Based

Carbon-based nanofillers are a wide group of compounds which includes single- and multi-walled nanotubes, graphite oxide, expanded graphite (EG), graphene, fullerene, and so forth [24,25].

The mode of action of carbon-based nanofillers is based on the formation of their char layer, which acts as a heat and thermal barrier [24]. However, carbon-based nanofillers are usually applied in combination with other FRs, due to the fact that they do not significantly improve the flame retardancy of the composite when used alone. The loading of these nanofillers is similar, in a range from 0.1 to 25 wt.% [23].

#### 4.2.1. Multi-Walled Carbon Nanotubes with Embedded Nickel Ferrite

The effect of NiFe_2_O_4_ deposited on the multi-walled carbon nanotubes (MWCNT) on the flammability of UPR nanocomposites was investigated by Yu et al. [36]. The nanofiller was synthesized via a chemical co-deposition method and annealing treatment and introduced into a UPR at a content of 2 wt.%.

The thermogravimetric analysis revealed the synergistic effects occurring between NiFe_2_O_4_ and MWCNT. High catalytic activity to carbonization of the former, as well as the regulatory effect in forming a compact network from MWCNT, resulted in a reduction in the maximum mass loss and formation of a protective barrier to block heat and mass transport. The char yield at 750 °C of pure UPR was 8.55 wt.%, whereas for the nanocomposite, it was 13.45 wt.%. Moreover, the nanocomposites had a lower intensity of gas emission during thermal degradation. The reduction in the evolved organic compounds realistically reduces both fire hazard and HRR. The pHRR was reduced from 1098 W/g (pure UPR) to 335 W/g (nanocomposite). The catalytic effect of NiFe_2_O_4_ is seen also in the oxidation of CO to CO_2_; thus, a reduction in emitted CO can be observed.

The reduction in HRR and THR was greater compared to pure UPR and composites containing separately used fillers, indicating a synergistic effect; however, these results also highlight the importance of achieving adequate dispersion of nanofillers in the matrix.

#### 4.2.2. Multi-Walled Carbon Nanotubes Coated with g-C_3_N_4_ Doped with Boron and Phosphorus

Although MWCNTs coated with g-C_3_N_4_ doped with B and P elements (BPCNT) did not show a significant improvement in the flammability of UPR materials, the synergism with APP was demonstrated by Chen et al. [37].

The thermal stability of UPR was slightly increased after the addition of BPCNT, and the temperature of the maximum weight loss was increased by 6 °C. On the one hand, the addition of APP reduced that value by 71 °C. On the other hand, the composite containing APP exhibited the highest char residue (31.0 wt.%). For the UPR modified with BPCNTs, it was slightly higher (12.0 wt.%) compared to that for pure UPR (8.9 wt.%). The addition of BPCNTs to a composite containing APP did not reveal any significant changes in the thermogravimetric analysis.

The synergistic effect between the APP and BPCNTs is seen in the LOI values. Modification of UPR by APP and BPCNTs improves LOI from 19.8% (pure UPR) to 29.0% and 22.3%, respectively. In contrast, the addition of both improves the LOI value to 30.6%. The highest decreases in pHRR, THR, SPR, and TSP were also observed for the composites containing both fillers.

Interesting results on the toughness of the composites were obtained. A significant decrease was observed for all composites. APP and BPCNTs decreased their values by 85.5% and 83.7%, respectively; however, a lower decrease in toughness was observed for UPR modified with both fillers (81.3%). A similar trend was observed for flexural strength.

#### 4.2.3. Pre-Expanded Graphite Container for Flame Retardants

The poor dispersion of EG in the UPR matrix, despite its great synergistic effect with phosphorus-based flame retardants, led to low effectiveness in reducing flame retardancy and reduced the mechanical properties of UPR materials. Hu et al. [38] developed a problem-solving approach by designing a nanocontainer for FRs based on pre-expanded graphite (pEG). The ionic liquid obtained from adenosine triphosphate (ATP) and 2-methylimidazole (MI) was encapsulated with pEG.

The addition of nanocontainers slightly improved the thermal stability of UPR. The one-step thermal degradation of pure UPR occurred with a maximum loss rate at 412 °C; in contrast, for UPR modified with EG and a 7 wt.% addition of nanocontainers, the temperatures were higher by 6 °C and 18 °C, respectively. The nearly double increase in the char residue at 700 °C, from 8.5 wt.% for pure UPR to 15.2 wt.% for UPR modified with nanocontainers, revealed the improvement in carbonization capacity.

A great increase in LOI was observed, from 23.8% for pure UPR to 31.0% and 33.0% for UPR modified with EG and nanocontainers, respectively, exhibiting the synergistic effects of EG, ATP, and MI.

The high pHRR of pure UPR (546 kW/m^2^) was decreased to 524 kW/m^2^ and 499 kW/m^2^ by the addition of EG and 7 wt.% of nanocontainers. However, the greatest decrease was observed for the 9 wt.% of nanocontainers (155 kW/m^2^). The TSP was also decreased, by 35% and 42%, respectively, for the UPR modified with 7 wt.% and 8 wt.% of nanocontainers.

The mechanical properties in terms of tensile strength were slightly reduced for modified UPR, from 27.9 MPa (pure UPR) to 25.4 MPa (UPR modified with 7 wt.% of nanocontainers). At the higher loading, the tensile strength was further reduced to 24.6 MPa.

### 4.3. Nanoscale Transition Metal Materials

**Figure 5:**
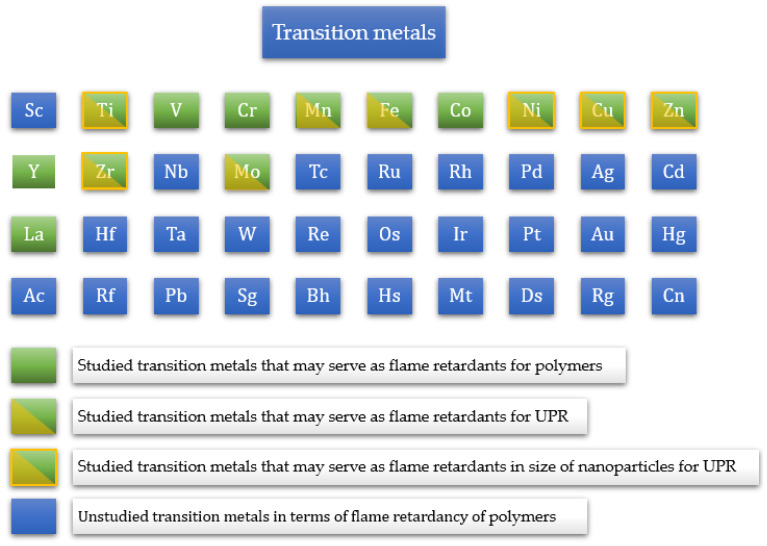
Overview of transition metals studied in terms of the UPR flammability [28,40,41,42,43,44,45,46,47,48,49,50,51,52].

The effect of transition metals on the thermal stability and flammability of polymers has not been studied in detail. However, due to their catalytic behavior, during burning, they may create C-C bonds resulting in the formation of a char layer, which makes them a potential group of FRs [39]. As shown in Figure 5, the current research mostly focuses on elements in the fourth period (from titanium to zinc), the fifth period (zirconium and molybdenum), and the sixth period (lanthanum). It can be seen that only titanium, nickel, copper, zinc, and zirconium, in the size of nanoparticles, have been studied so far in terms of their influence on the thermal stability and flammability of UPRs.

#### 4.3.1. Zinc(II) Oxide

Due to the hydrophilic nature and high polarity of ZnO nanoparticles, their surface needs to be modified to achieve better compatibility and adhesion with the nonpolar and hydrophobic polymer matrices. Chen et al. [48] studied the thermal stability and tensile strength of UPR nanocomposites modified with zinc oxide(II). Nanoparticles were prepared by grafting the zinc oxide by aminopropyltriethoxysilane (APS) and oleic acid (OA) activated by N,N′-carbonyldiimidazole.

Up to 425 °C, the weight loss of the UPR/ZnO nanocomposite was higher compared to pure UPR, achieving its highest value at 365 °C (0.75 mg/°C). Above that temperature, the fragmentation of oleic acid occurs, resulting in the improvement of thermal stability, which can be seen in the decrease in the weight loss rate compared to pure UPR (1.06 mg/°C at the temperature of 375 °C). The residue at 600 °C of pure UPR (97%) is significantly higher in comparison to that of the nanocomposite (83%).

The tensile strength of the pure UPR (20 MPa) majorly increases up to 40 MPa for a nanocomposite containing 3 wt.% of the nano ZnO. Further increase in the nanoparticles content results in the decrease in tensile strength to 26 MPa (10 wt.% of nano ZnO). The bending strength exhibits similar behavior; however, its increase is slightly lower. The increase in tensile and bending strength may be ascribed to the fact of sharing stress with long chains of oleic acid, as well as the incorporation of ZnO nanoparticles into the UPR structure by chemical bonding.

#### 4.3.2. Cuprous(I) Oxide

Hou et al. [49] studied the effect of different sizes of Cu_2_O particles on the thermal and combustion behaviors of UPR. Cu_2_O of different sizes (10 nm, 100 nm, 200 nm) was synthesized by the wet colloidal method from a copper sulfate with the usage of polyvinyl pyrrolidone.

The thermogravimetric analysis revealed a two-stage thermal decomposition process of pure UP and nanocomposites, with the first step ascribed to the degradation of chains and the second step ascribed to the char residue oxidation. The second stage of decomposition for the pure UPR stars at 500 °C; in contrast, for the UPR/Cu_2_O nanocomposites, it occurs at lower temperatures, in a range from 450 °C (Cu_2_O particle size of 10 nm) to 460 °C (Cu_2_O particle size of 200 nm). The char yield at 750 °C of all UPR/Cu_2_O nanocomposites is higher than that of pure UPR and increases with the increase in Cu_2_O particle size.

The highest value of pHRR, 600 W/g, measured using a microscale combustion calorimeter, was obtained by a nanocomposite containing the Cu_2_O of particle size of 10 nm; this reflects the pattern of the thermogravimetric curve and promotion of the degradation of UPR by the catalytic effect. The decrease in pHRR was observed for nanocomposites containing Cu_2_O of the size of 100 nm and 200 nm (a decrease of 21.5% compared to pure UPR). This results in a conclusion that, with the increase in the particle size, the barrier effect enhances over the nanometer effect; this may be ascribed to the fact that particles with greater size more firmly settle in the matrix and form networks during the pyrolysis of UPR, promoting the formation of a char layer.

In addition, the steady-state tube furnace (SSTF) test results showed that the addition of Cu_2_O to UPR significantly increases the CO_2_ yield and decreases the CO yield, which positively affects fire safety in terms of evacuation. The greatest improvement is observed for the smallest Cu_2_O size, due to its thermo-catalytic performance, which was better than that of the other specimens.

#### 4.3.3. Nanorods Containing Nickel(II)

The effect of micro/nanorods structured with diphenylphosphinyl groups, Schiff base, and nickel(II) on the thermal stability and mechanical properties of UPR was investigated by Li et al. [50]. The three-step synthesis of [1,2-phenylenebis(azanediyl)-bis(2-hydroxyl-5-diphenylphosphinylphenylmethylene)] nickel(II) (SDPPNi) is based on the self-assembly coordination reactions of Salen S, diphenylphosphinic chloride, and nickel(II) acetate.

Similarly to UPR/Cu_2_O, the UPR/SDPPNi nanocomposites exhibit a two-stage decomposition process, with the second step starting at lower temperatures (470 °C–480 °C) for nanocomposites compared to those required for pure UPR (540 °C). The temperature at the maximum mass loss rate decreases with the increase in SDPPNi content in both degradation stages, from 415 °C and 563 °C for UPR to 399 °C and 494 °C for the UPR/SDPPNi containing 25 wt.% of the additive. It is ascribed to phosphoric acid, formed by the decomposition of diphenylphosphinyl groups, which promoted degradation processes. The increase in the residue yield of 4.8 wt.% compared to pure UPR, with the increase in SDPPNi content, suggested that it enhances the formation of char.

LOI tests revealed that the addition of SDPPNi significantly improves the LOI value, from 18% for pure UPR to 38% for UPR/SDPPNi containing 25% of the additive. The pHRR value at 10 wt.% of additive was reduced by 50% compared to pure UPR, and the increase in the SDPPNi content further decreased the pHRR values. Therefore, the THR also decreases. The peaks of the carbon monoxide production rate follow the same decrease trend. The peak CO production for pure UPR (0.106 g/s) decreases by 37% for the UP/SDPPNi containing 10 wt.% of additive and by 66% for the nanocomposite containing 25 wt.% of SDPPNi.

The smoke density of UPR is important in terms of evacuation and for its potential applications in the shipbuilding, construction, and transportation industries. The smoke optical density D_s_ (-) is also improved for the nanocomposites. The D_s_ maximum value for the UPR of 1320 decreases up to 769 for the nanocomposite with 25 wt.% additive of SDPPNi.

Although the greatest improvement of the thermal stability and the flammability parameters had been achieved for the UPR containing 25 wt.% of SDPPNi, the amount of additive is tremendously higher compared to other nanoparticles. However, due to the uniform dispersion of SDPPNi in the UPR matrix, a significant reduction in mechanical properties is not observed. For the UPR containing 10 wt.% of additive, the tensile strength (53.1 MPa) was reduced by only 0.1 MPa compared to that of pure UPR. For the highest amount of additive, it is reduced by 19% (43.1 MPa). A slightly higher decrease in the impact strength is noted for nanocomposites, from 6.69 kJ/m^2^ for pure UPR to 5.07 kJ/m^2^ for UPR/SDPPNi (25 wt.%).

#### 4.3.4. Titanium(IV) Oxide

The effect of the commercially available nano titanium oxide on the flammability and smoke suppression properties of UPR was investigated by Zatorski et al. [51]. The addition of the TiO_2_ reduces the pHRR from 820.96 kW/m^2^ for the pure UPR to 530.95 kW/m^2^. The THR was reduced only by 7%. However, the authors compared several nanocomposites containing carbon nanotubes, aluminosilicates, and polyhedral oligomeric silsesquioxanes, and the obtained reduction in THR was the highest among all the tested materials. Research revealed that the fire growth rate (FIGRA) of pure UPR (2.93 kW/m^2^ s) was reduced by 55% to the value of 1.30 kW/m^2^ s, exhibiting the greatest improvement.

The nano TiO_2_ appears to have smoke suppression properties: the D_s_ maximum value of nanocomposite was 638, which is 17% lower compared to pure UPR (773). The results of the smoke optical density were in agreement with the results on the decrease in the total smoke released (TSR).

#### 4.3.5. Allylamine-Exfoliated Alpha Zirconium Phosphate

Pichaimani et al. [52] studied the influence of allylamine-exfoliated alpha zirconium phosphate (AZrP) on the flammability and mechanical properties of UPR. The nano FR was prepared by the Brønsted acid–base interaction.

The temperatures of the 5 wt.% of mass loss during thermal degradation for nanocomposites containing 2 wt.% and 5 wt.% of AZrP were lower (133 °C and 125 °C, respectively) compared to pure UPR (172 °C), which corresponds to the release of water absorbed by composites. Although the nanocomposites containing 7.5 wt.% and 10 wt.% of additives exhibited a 5 wt.% mass loss at higher temperatures, the main degradation process occurred at higher temperatures (400 °C) compared to pure UPR (300 °C) for all nanocomposites. The highest residue, obtained at 700 °C, was obtained for the 10 wt.% of additive (23.9 wt.%).

The LOI value for pure UPR (17.5) was significantly lower compared to nanocomposites. The flammability decreased with the increase in additive content. The highest LOI was obtained for the UPR containing 10 wt.% of AZrP (27.1), revealing its self-extinguishing behavior. The tests reveal that AZrP, due to its uniform dispersion, may act as a heat barrier, resulting in the decrease in heat radiation into a polymer matrix.

The positive effect on the mechanical properties was noticed for the nanocomposites containing 5 wt.% of additives or more. The greatest improvement in tensile strength and flexural strength was observed for the 10 wt.% of additives (59.4% and 56.7%) compared to pure UPR. This may be ascribed to the transfer of stress to the layered hexagonal AZrP, and the presence of flexible chains, thus improving the tensile strength and flexural strength of nanocomposite.

#### 4.3.6. Ti_3_C_2_Tx (MXene) Nanosheets

Max phases, being layered hexagonal carbides or nitrides, take the name from their general formula M_n+1_AX_n_ (n—1–3), where M stands for the early transition metal, A stands for the main group element, and X stands for the carbon or nitrogen. They are precursors for the MXene, studied by Hai et al. [53], who investigated its effect on the flammability of UPR.

MXene, with a general formula of Ti_3_C_2_T_x_, where T_x_ stands for their surface termination groups, was synthesized by the hydrofluoric acid etching of MAX, resulting in the removal of all Al elements, an increase in the interlayer spaces, and the emergence of new terminations of -OH, =O, and -F groups.

A considerable improvement in flammability properties was observed for the nanocomposites. The pHRR value of pure UPR (743.19 kW/m^2^) was reduced by 29.6%, to a value of 523.47 kW/m^2^, and the THR was reduced by 14.8% to a value of 85.50 (kW/m^2^). Moreover, the total smoke production (TSP) was decreased from 18.45 m^2^ (for the pure UPR) to 13.79 m^2^ for the nanocomposite. Furthermore, the carbon monoxide production was decreased (by 31.6%), as was the carbon dioxide production (by 27.9%), indicating the great smoke-suppression properties held by MXene via the physical barrier effect. The char residue of pure UPR (0.78%) was significantly lower than that for the UPR/MXene nanocomposite (5.04%).

The deep crosslinking and great dispersion of MXene in UPR results in an increase in the tensile strength from 37.01 MPa for pure UPR to 41.6 MPa. However, the elongation at the break is slightly reduced from 8.1% to 7.4%, which shows the weaker plastic strain capacity of the UPR/MXene nanocomposite.

#### 4.3.7. Cu_2_O–TiO_2_–Graphene Oxide Dual Nanosheets

The synthesis of the Cu_2_O–TiO_2_–graphene oxide (GO) nanosheets via the hydrothermal reaction with graphene oxide, tertbutyl titanate, and copper acetate, and its effect on the flammability of UPR was investigated by Wang et al. [54].

The thermal analysis of nanocomposites revealed that the 2 wt.% addition of functionalized GO nanosheets has a negligible effect on the thermal stability of UPR. The char residues of TiO_2_/UPR, Cu_2_O–TiO_2_/UPR, and Cu_2_O–TiO_2_–GO/UPR at 800 °C were only slightly higher, and the onset temperatures of the two stages of thermal decomposition closely coincided with those obtained for the pure UPR.

However, the effect of the Cu_2_O-TiO_2_-GO on the flammability is noticeable. The pHRR of the Cu_2_O-TiO_2_-GO/UPR (631 kW/m^2^) was reduced by 29.7% compared to pure UPR (897 kW/m^2^). A significant reduction in THR is observed from the 59.2 MJ/m^2^ (pure UPR) to 47.9 MJ/m^2^, as well as the FIGRA decrease by 46%.

### 4.4. Layered Double Hydroxides (LDHs)

Layered double hydroxides (LDHs), with a general formula of [M^2+^_1−x_M^3+^_x_(OH)_2_]^x+^A_x/n_^n−^·H_2_O, are built of brucite-like layers of metallic cations (M) and hydroxyl groups, intercalated with inorganic or organic anions (A) and solvation molecules which compensate for their positive charge. Due to their adjustable structure, when properly selected metals and anions, they may serve as ecofriendly FRs [23,24] and smoke suppressants; they can also be used as absorbers, drug delivery hosts, precursors, or catalysts [55].

The mode of action of LDHs is based on the enhanced stable char layer formation, reducing the accessibility of fuel, and the dilution of the concentration of combustible gases by releasing water molecules. The loading of this nanofiller is similar to clay-based nanoparticles and is in a range of 0.1 to 10 wt.% [23].

#### 4.4.1. Dodecyl Sulfate Intercalated Magnesium Aluminum Nitrate LDH

Due to the fact that inorganic hydroxides, such as Al(OH)_3_ or Mg(OH)_2_, are effective as FRs when used in high dosage, materials such as LDHs—being more compatible with the polymer matrix and obtaining greater dispersion due to lower hydrophilicity—have become a good choice for obtaining a proper level of flame retardancy. The lower amount of LDH is needed to achieve the same physical barrier effect; however, it must be used in combination with other FRs. Kaul et al. [56] studied the effect of trixylenyl phosphate (TXP) and dodecyl sulfate intercalated with magnesium aluminum nitrate LDH (MgAl DS LDH). The nano FR was synthesized via the co-precipitation method and ion exchange.

The results revealed that the addition of MgAl DS LDH to the UPR containing TXP leads to an increase in the thermal stability of the nanocomposite. The decomposition of LDH to water and metal oxides catalyzes and enhances the formation of char from fragmented polymer chains. The greatest thermal stability was obtained for nanocomposite containing 24 wt.% of TXP and 1 wt.% of MgAl DS LDH, the temperature at weight loss of 70 wt.% was the highest (393.9 °C), and the residue yield at 600 °C was the highest (5.43 wt.%). Further increase in the LDH content and decrease in the TXP content results in a decrease in thermal stability.

#### 4.4.2. Nickel Iron Nitrate LDH

The growth of LDH on fabric sheets helps to avoid the agglomeration of LDH in the nanocomposite. This approach was adopted by Chu et al. [57]. NiFe LDHs grown on ramie fabric and coated with phosphorus and silicone coating significantly improved the thermal and mechanical properties of nanocomposites. The functionalization of fibers improved heat dissipation, which resulted in higher thermal stability of UPR/ramie fabric/LDH composites. Composites containing P and Si coating had the highest residue yield, which confirms the synergistic effects between LDH and phosphorus coatings. The usage of functionalized fabrics influences fire safety. The heat release rate (HRR) of UPR/ramie fabric/LDH and UPR/ramie fabric/LDH/PSi composites was significantly reduced by 28.17% and 36.56%, respectively. Although the functionalization of fabric decreases the THR of the composite compared to a composite containing nonmodified fabric, only the application of coating reduces its value by nearly 50%. Moreover, the functionalization of fabrics and the subsequent coating application positively affects the mechanical properties of nanocomposites. The tensile strength of UPR/ramie fabric composites was 87.81 MPa; in contrast, after the functionalization of fabrics and/or the application of the coating, the values increased to 100.16 MPa and 103.77 MPa, respectively.

### 4.5. Polyhedral Oligomeric Silsesquioxanes

Polyhedral oligomeric silsesquioxanes (POSSs) are hybrid inorganic–organic materials whose structures may be polyhedral or cage-like, containing a silicon–oxygen core and substituents on the corners. The thermal and chemical robustness of these materials is given by the inorganic framework, and other properties may be conferred by organic substituents, which may be reactive or not. In addition to an increase in flame retardancy, nanocomposites containing POSS exhibit lower viscosity, greater mechanical properties, and oxidation resistance, among others [58].

#### 4.5.1. POSS-Functionalized Graphene Oxide

Divakaran et al. [59] studied the effects of POSS-functionalized graphene on the flammability of UPRs. Graphene oxide (GO) was functionalized by POSS-containing NH_2_ groups by forming peptide bonds, with a dicyclohexylcarbodiimide as a catalyst.

Nanocomposites containing from 0.05 to 0.3% of GO/POSS exhibited lower weight loss at the second degradation step concerning pyrolysis when compared to pure UPR. A significant increase in the temperatures of the 10 and 50 wt.% weight loss was observed, and the highest increase was obtained for the nanocomposite containing 0.3 wt.% of GO/POSS (26.9% and 7.3%, respectively). Breaking the polymer bonds in pure UPR occurred at ca. 350 °C, and for nanocomposites, the main thermal degradation step occurred at higher temperatures. Therefore, the mode of action of GO/POSS is based on the barrier effect, blocking mass, and heat transfer.

LOI values of nanocomposites were higher compared to pure UPR (22 vol.%). The increase was observed with the increase in the content of additive, but only up to 0.1 wt.% of POSS/GO (25 vol.%). Nanocomposites containing a greater amount of additive (0.3 wt.%) exhibited a decrease in the LOI value, due to the nanoparticles’ tendency to aggregate, leading to a lower quality of the formed barrier.

The improvement of tensile strength compared to pure UPR was observed for nanocomposites due to the transfer of stress loadings from the polymer to the nanofillers. However, the increase in the tensile strength was also observed for up to 0.1 wt.% (61.9%) of GO/POSS. Its further addition resulted in a decrease in tensile strength due to the agglomeration of nanoparticles inducing local stress concentrations.

#### 4.5.2. POSS-Modified MMT

The POSS-NH_2_ modification of MMT was proposed by Divakaran et al. [60], by the intercalation of POSS between the MMT layers.

Thermal analysis of nanocomposites containing 0.5, 1, 3, and 5 wt.% of POSS-MMT revealed that both temperatures of the 10 and 50 wt.% of weight loss increased with an increasing amount of the additive, and the highest increase was observed for the composite with 5 wt.% of nanofiller (by 14.3% and 7.03% compared to pure UPR). Similarly to previous research (Section 4.5.1), with the addition of POSS/MMT—due to the high bond energy of Si-O bond and stiffness of the framework of the nanofiller—the main degradation step was shifted towards higher temperatures.

Although the addition of POSS/MMT results in an increase in the tensile strength, the greatest value was observed for the nanocomposite containing 3 wt.% of the additive. Further increases in the POSS/MMT do not induce further significant increases in tensile strength. The dispersion of nanoparticles is therefore crucial, and the agglomeration of nanoparticles such as POSS/MMT and POSS/GO needs to be below the critical concentration.

#### 4.5.3. POSS-Modified Octamaleimide

Thermal properties of POSS-modified octamaleimide and UPR hybrid nanocomposites were studied by Jothibasu et al. [61]. Octa(maleimido phenyl) silsesquioxane (OMPS) was added in order to form a highly crosslinked network. Due to that fact, and the presence of a stable maleimide group, the degradation of the nanocomposites started at higher temperatures as compared to pure UPR. While the decomposition temperature of the unmodified resin was 334 °C, a 1 wt.% addition of OMPS increased the value to 386 °C, and a 10 wt.% addition increased it to 397 °C. An almost threefold increase in char yield was also observed for nanocomposites containing 10 wt.% of OMPS (22.3 wt.%) compared to pure UPR (8 wt.%).

### 4.6. Others

The current need for the usage of renewable and sustainable materials pushes research towards the synthesis of bio-based FRs. The biomass conversion leads to the formation of four fractions: carbohydrates, proteins, lipids, and phenolic compounds. Saccharide-based products (cellulose, starch, and chitosan), bio-based aromatic compounds (lignin, tannins, gallic and ellagic acids, DNA), and proteins (casein, phytic acid) are some examples of compounds that may serve as FRs as they are or after modification. The mode of action of most of these compounds focuses on their char-forming ability [62]. Other examples of bio-based nano FRs are calcium carbonate, cyclodextrins, and hydroxyapatite [23]. Among these bio-based compounds, some cellulose-based compounds can be obtained in the nanoscale: nanofibers and nanocrystals may be used in order to improve the thermal stability of composites [25]. However, to date, bio-based nano FRs used for UPR in order to improve its thermal stability have been poorly researched.

In addition to nano bio-based FRs, there are also reports on the use of volcanic rocks, such as pumice, or nitrides to improve the thermostability of UPRs.

#### 4.6.1. Nano-Active Modified Pumice

Rakhman et al. [63] studied the effect of the active modified pumice nanoparticles in combination with aluminum trihydroxide (ATH), sodium silicate (SS), and boric acid (BA) on the thermal and mechanical properties of glass/polyester laminate.

Nanoparticles were synthesized via the sol-gel method. The silica-rich pumice was prepared from thermally activated pumice and then dissolved in NaOH to produce sodium silicate. The silica gel was obtained by filtering in ethanol and HNO_3_. The nano-active modified pumice (nAMP) was added into the matrix in a mixture with ATH, SS, and BA.

The thermogravimetric analysis revealed the two-step decomposition of pure glass/polyester laminate. The replacement of the 10 wt.% of the UPR by the mixture containing 1 wt.% of nAMP resulted in a decrease in the mass loss at the second degradation step and an increase in the residue weight at 600 °C by more than 10 wt.%. The DSC curves and combustion tests showed that modified composites exhibit greater ignition time (106 s) compared to nonmodified (81 s) ones, and that more heat is needed to start the decomposition. The exothermic peak corresponding to the thermal oxidation of released volatiles occurs at 352 °C; in contrast, for the pure composite, it occurs at 346 °C, showing the retardment of the oxidation.

Although the impact strength of modified composites increased by 15.4%, the flexural strength was significantly reduced (from 45.48 MPa to 39.81 MPa). However, increasing the content of nAMP in a mixture leads to an increase in both the studied mechanical properties. The greatest increases in the flexural strength (28.6%) and impact strength (34.9%) were achieved when the nAMP content was increased to 4 wt.%. This results in the conclusion that, although the higher content of the nAMP positively affects the mechanical properties, it simultaneously reduces the FR properties of a modified composite.

#### 4.6.2. Boron Nitride Nanosheets

Wang et al. [64] studied the effect of functionalized boron nitride nanosheets on the flame retardancy of the UPR. The boron nitride nanosheets co-containing phosphorus, nitrogen, and silicon elements were synthesized via the high-temperature annealing process and ultrasonic hydrolysis of boron nitride, its amination with the (3-aminopropyl)triethoxysilane, and further Michael addition reaction with hyperbranched polyphosphate acrylate (HPPA), containing phosphorus and nitrogen.

The obtained cone calorimeter test results show that the functionalization of boron nitride nanosheets positively affects the pHRR values. Additions of 1 and 3 wt.% decrease the pHRR from 1211 kW/m^2^ for pure UPR to 1034 kW/m^2^ and 870 kW/m^2^, respectively. Further increases in the amounts of additives only slightly decrease the pHRR value (829.54 kW/m^2^), which is ascribed to the aggregation of nanosheets. However, the 3 wt.% addition of non-functionalized nanosheets to UPR marginally decreases the pHRR by 2.6%, which brings the more effective functionalized structure to our attention. The THR values of functionalized boron nitride nanosheets were lower compared to those of pure UPR (65.3 MJ/m^2^), and the greatest improvement was also observed for the nanocomposite containing 3 wt.% of additive (40.5 MJ/m^2^). The CO and CO_2_ yields were lower for all nanocomposites, due to the nano-barrier effect of nanosheets. The best results were obtained for the additive of the amount of 3 wt.%.

The initiation of the thermal degradation of UPR containing the functionalized nanosheets occurs at a temperature lower by 68 °C compared to pure UPR, which is associated with the degradation of the phosphorus groups of HPPA. However, the nano-barrier effect and the catalytic carbonization of the HPPA positively affect the maximum mass loss rate values.

## 5. Environmental Impact of Nano Flame Retardants and Nanocomposites

### 5.1. Life Cycle Assessment

Recently, Carroccio et al. [65] critically reviewed life cycle assessment (LCA) studies concerning nanocomposites and nanoparticles. LCA is a well-known method for the assessment of the environmental impacts of materials, and their results may reveal that petroleum-based plastics, which have well-established and properly designed recycling processes, are more sustainable than bio-based plastics, due to their higher energy consumption or the high cost of raw materials.

Although nanofillers constitute merely a fraction of nanocomposites, their synthesis and modification can be a major environmental burden, and can significantly affect the overall environmental impact of the nanocomposite, The assumption that a small amount of nanofiller in a nanocomposite does not significantly affect the overall environmental impact may turn out to be misleading.

LCA takes into account both the necessary amounts of reagents for the given synthesis or modification processes of nanoparticles and their excess if must be used. This may be the reason for the large differences in results obtained by different authors. The approach shows that the type of process, its efficiency, the cost of raw materials for synthesis, and further functionalization to obtain appropriate dispersion in nanocomposite affect the total values of the various impact categories, such as nonrenewable energy use (NREU) or global warming potential (GWP).

It might seem that bio-based nanoparticles will not significantly affect the environment. However, as in the case of nanocellulose, the cultivation of plants involves heavy use of water and fertilizers, and the biomass conversion and extraction processes are highly expensive [66].

In some cases, e.g., carbon nanofibers, savings that came from the decrease in nanofiller loading can outweigh the energy costs required for nanocomposite production, justifying manufacturers’ choice of the traditional solution.

The historic use of selected nanofillers and the consequent high technological readiness level of the process mean that the production of nanoparticles such as titanium oxide may have a slightly lower environmental impact than others.

The authors divided nanoparticles into three groups according to the environmental impact of nanocomposite production: (1) nanoparticles whose addition shows no additional impact of the nanocomposite on the environment—e.g., clay, carbon black, graphene; (2) nanoparticles that increase the environmental burden by about ten times—e.g., carbon nanofibers, nanocellulose, TiO_2_, Ag; (3) nanoparticles that significantly affect the environmental burden and increase impact categories such as NREU and GWP by two and four orders of magnitude, respectively—e.g., carbon nanotubes [65].

### 5.2. Effects on Human Health

The potential toxicity of nanoparticles is related to their ability to penetrate soil, water, and air. Artificially produced nanoparticles may contain stabilizers and surfactants, making them more prone to accumulation. The assessment of their potential toxicity needs to include all processes, including production, transport, usage, and end-of-life management.

During the production step, it is necessary to strictly control the occurring processes in order to avoid nanoparticle emission, which may be direct (e.g., emission of powders through open windows) or indirect (inappropriate waste treatment).

During the usage of nanocomposites, intentional or accidental release of nanoparticles may occur, with the rate of emission depending on the type of product. From solid products, such as UPR nanocomposites, the emission will be slower compared to fluid or spray products [67].

The recycling process of glass-fiber-reinforced UPRs remains an ongoing issue. Mechanical recycling, involving the shredding or grinding of waste GFRP and their further application as a part of reinforcement, reduces their mechanical properties [68]. Other methods, such as fluidized bed combustion or chemical recycling, enable the recovery of glass fibers only, seldom exhibiting the same mechanical properties as before. Landfilling of such non-recoverable waste remains the only method of dealing with this type of material [69].

On one hand, during landfilling, the emissions of nanoparticles may or may not occur. On the other hand, incineration may result in the presence of nanoparticles in ash, but not in the air, due to the high efficiency of filters.

Although quantitative analysis of the nanoparticles emitted during the nanocomposite manufacturing stage provides meaningful results, determining their content concerning emissions at other stages of the nanocomposite life cycle remains a challenge, due to the transformations they may undergo. These transformations include photochemical changes, redox processes, dissolving and precipitating, sorption or desorption processes, combustion, biotransformation, or abrasion. As a result, the toxicity of nanoparticles should be adequately tested [67].

Generally, there remains a lack or a small amount of data regarding potential hazards posed by nanoparticles, especially at the end-of-life stage. During toxicity and health effects studies, the phenomenon of releasing smaller particles formed during combustion, the ability to travel large distances, and the increases in the sediment and accumulation processes in the lungs have to be taken into consideration [70].

The cellular exposition to nanoclay may result in mitochondrion damage, a decrease in cell proliferation, the generation of reactive oxygen species, and even damage to the DNA. Research conducted by Wagner et al. [70] revealed that nonmodified nanoclay and its thermally degraded products exhibit lower cellular toxicity compared to modified nanoclay.

Although well-functionalized carbon nanomaterials are safe, nonmodified ones exhibit high toxicity to human and animal cells. Despite reports on carbon nanomaterials inhibiting the proliferation of tumor cells, health hazards have not yet been studied in depth [71].

During the dissociation of metal oxide nanoparticles, metal ions are released, which are mainly responsible for their toxic potential. These oxides can cause genotoxicity, cytotoxicity, and immunotoxicity. Copper oxide nanoparticles entering the respiratory tract can cause inflammation. They can penetrate further into the body or accumulate in the lungs, causing the generation of reactive oxygen species and oxidative stress. Oral exposure can expose people to hepatotoxic effects and ulcer formation [72].

Titanium dioxide is classified by the International Agency for Research on Cancer as a suspected carcinogen. Recently, the European Commission decided to ban its usage in the food industry. Oral ingestion or inhalation results in the accumulation of nano TiO_2_ in digestive, respiratory, and reproductive system structures. Moreover, it may adversely affect the development of the ovum, and further influence the health of offspring [73].

Among various nanoparticles, LDHs exhibit less toxicity than other nanoparticles. Their biocompatibility allows them to be used as drug nanocarriers [74]. However, sudden side effects such as death, caused by intravenous injection of LDHs—due to their aggregation when exposed to physiological fluids—indicate the need for more detailed research [75]. POSSs also have low toxicity and exhibit biocompatibility; thus, they may be used in bone regeneration or drug delivery systems [76].

Depending on the physicochemical properties, such as size, porosity, shape, and surface, silica nanoparticles can affect human health in various ways. Silica nanoparticles may enter the respiratory tract and accumulate in the alveoli. Their inhalation can cause silicosis. When not secreted by microphages, they can cause lung tissue damage or pass further into the body through the bloodstream. Delivered via the gastrointestinal tract, they can be deposited in the lipid layers of organs [77].

Nanocellulose (NC) can potentially persist in the lungs, and its inhalation has a deleterious effect on human health. Studies on the dermal and oral toxicity of NC did not reveal any negative effects. However, inconclusive results have emerged from cytotoxicity and pulmonary studies. Long-term studies in terms of exposure via the pulmonary route and dermal contact are underrepresented in the literature [78].

All mentioned environmental and health effects of nano FRs used for UPR have been summarized in Table S3.

### 5.3. Fire Hazard

The change in the size scale of fillers from micro to nano should also take into consideration safety concerns during handling, transport, and storage.

After a reduction in the size of fillers, e.g., the change in the size of metal powders from micro to nano, we can observe drastic changes in their magnetic and electrical properties. However, due to the enhancement of the surface area, we can observe also major changes in their explosivity. This reduction, therefore, may result in increased susceptibility of their powders and dust clouds to ignition [79].

## 6. Conclusions

The potential of nanoparticles has been exploited in various industry fields, including the pharmaceutical, microelectronics, aerospace, food processing, packaging, energy, and engineering industries, depending on their physicochemical properties. Although the low toxicity of these materials has been claimed for many years, nowadays, more and more researchers are paying attention to their potential toxic effects on humans and the environment. It turns out that it is only recently that in-depth toxicity studies have been conducted that also take into account the transformations that nanoparticles undergo in the environment and the human body.

Several groups of nanoparticles may serve as excellent FRs in application in UPRs, with significantly lower loading compared to traditional FRs.

Although the size of nanoparticles—and thus high surface area/volume ratio—accounts for their effectiveness, it has been observed that the size of nanoparticles may also change their mechanisms of action, as is the case for Cu_2_O.

On the one hand, the addition of nanoparticles of up to 10 wt.% usually improves the mechanical properties of the composite, such as tensile and impact strength. On the other hand, some of them may slightly decrease their values at high loadings (from 10 to 25 wt.%). However, the nonsignificant decline in mechanical properties can occur simultaneously with a high improvement in thermal stability, such as for nickel-containing nanorods.

The content of nanoparticles in nanocomposites is very important. The increasing amount of the additive usually improves thermal stability, but this phenomenon is usually observed up to a certain content of nanoparticles, above which their agglomeration increases, and their thermal stability does not improve as much as it would appear; this is the case for POSS/MMT and POSS/GO.

Environmental issues concerning the use of nanoparticles as FRs are also very important. The emission of nanoparticles can occur at different stages of the composite’s life, and when separated into the environment, they can transform, making it difficult to realistically assess their emissions. Legislation should be carried out in such a way as not to hinder the further development of nanotechnologies, but should take into account the latest research on their toxicity. It is also worth noting the environmental burdens that are created by the modification of nanoparticles to achieve proper dispersion in the UPR matrix.

Due to the fact that most nano FRs do not significantly affect mechanical properties, future research should focus on those groups of compounds which exhibit great enhancement of thermal stability of composite while also not posing a threat to the environment or human health. Based on the results of this study, the most promising groups of nano FRs are LDHs, POSS, and carbon nanomaterials, with the proviso that their production methods will be optimized with respect to energy consumption from nonrenewable raw materials.

## Data Availability

No new data were created or analyzed in this study. Data sharing is not applicable to this article.

## Supplementary Materials

The following supporting information can be downloaded at: https://www.mdpi.com/article/10.3390/ma17040852/s1, Table S1: Effects of the application of nano flame retardants in unsaturated polyester resin. Table S2: Combustion characteristics of nano FRs with regard to pure UPR. Table S3: Environmental and health effects of nano flame retardants in unsaturated polyester resin.
