# Supplementary material for: Recent Developments of Nano Flame Retardants for Unsaturated Polyester Resin
Source: Materials (Basel). 2024 Feb 11;17(4):852. doi: 10.3390/ma17040852 (PMC10890331; doi:10.3390/ma17040852)
Supplement: Supplementary file 1 [file materials-17-00852-s001.zip › materials-2772448-supplementary.pdf]

materials

MDPI

Supplementary Materials: Table S1, Table S2, Table S3.

Table S1. Effects of the application of nano flame retardants in unsaturated polyester resin.

Nanoparticle	Form	Synthesis method	Content	Size	Mode of action	Effect on the thermal stability and flammability	Effect on the mechanical properties	Reference
Organic-modified montmorillonite with 25-30 wt. % of methyl dihydroxyethyl hydrogenated tallow ammonium	Nanosheet	Not investigated	1, 3, 5 wt.% (3 wt.%—optimal)	Lateral dimension—200-300 nm; thickness—1 nm	Not investigated	Decrease in the pHRR; decrease in FGI; decrease in TTI	Not investigated	[1]
Ionic liquid functionalized imogolite nanotubes (as a synergist to APP)	Nanotubes	Arancibia–Miranda synthesis of imogolite with tetraethoxysilane, aluminum nitrate nonahydrate, and ammonia; ionic liquid functionalization via the Wan method with the 1-butyl-3-methylimidazolium hexafluorophosphate and 3-aminopropyltriethoxysilane	0.4 wt.% (16.6 wt.% of APP)	Length—from 100 nm to 200 nm; diameter—from 10 nm to 20 nm	Promoting char layer formation blocking heat and oxygen transfer	Higher temperatures of the second and the third degradation steps; increase in the residue yield at 730 °C; improve LOI	Not investigated	[2]
MWCNT/ $NiFe_2O_4$	Not specified	Chemical co-deposition method and calcination	2 wt.%	Not specified	Barrier effect, blocking heat, and mass transfer	Decrease in the temperature of the 10 wt. % of mass loss; increase in the char yield at 800 °C; reduction in emitted volatile compounds, including CO; reduction in pHRR and THR	Not investigated	[3]
MWCNTs coated with $g\text{-}C_3N_4$ doped with B and P, with APP	Not specified	One-pot hydrothermal synthesis	2 wt.% of BPCNTs, 18 wt.% of APP (18 wt.%	15.7 nm	The formation of a glassy layer increases the char yield; catalysis of the formation of aliphatic compounds and phosphate esters and enhancement of formation of com-	Significant decrease in LOI; significant decrease in HRR, THR, TSP, and SPR	Significant decrease in flexural strength and toughness	[4]

Nanoparticle	Form	Synthesis method	Content	Size	Mode of action	Effect on the thermal stability and flammability	Effect on the mechanical properties	Reference
			of APP when used alone)		bustible gaseous substances; capturing free radicals; MWCNTs promote the formation of the dense carbonaceous layer preventing air and heat exchange			
pEG nanocontainer for FRs	Mesopore structure (pEG)	Encapsulation of ATP and MI into pEG	7, 9 wt.% (7 wt.%—optimal)	3.07 nm (pEG pore diameter)	Promotion of the formation of the char layer; release of phosphorous-containing volatiles capturing free radicals	Slight increase in the thermal stability; increase in the residue yield at 700 °C of 6.7 wt. %; significant increase in LOI by 10%; decrease in pHRR and THR; decrease in TSP and SPR	Slight decrease in the tensile strength	[5]
Zinc(II) oxide ZnO	Not specified	Grafting of the zinc oxide by aminopropyltriethoxysilane (APS) and oleic acid (OA) activated by N,N′-carbonyldiimidazole	1, 3, 5, 10 wt.% (3 wt.%—optimal)	Not specified	Not investigated	Increase in the thermal stability above 365 °C; increase in the residue yield at 600 °C of 15 wt. %; significant increase in the tensile and bending strength	Significant increase in tensile and bending strength	[6]
Cuprous(I) oxide Cu_2O	Nanosphere	Colloidal synthesis from copper sulfate with polyvinyl pyrrolidone	Not specified	10 nm (S) 100 nm (M) 200 nm (L)	Acceleration of the degradation of UPR groups containing oxygen, thus generation of more H_2O, CO_2, and gaseous products increasing the HRR; the Cu_2O (S) particles contribute to complete combustion; for the Cu_2O (M) and (L), the superficial area increases; the char layer formation gradually subsides the catalytic effect; thus the combustion suppression is achieved	Decrease in the second degradation step temperature of 50 °C; increase in the residue yield at 750 °C; decrease in the pHRR (when Cu_2O particle size is 100 nm or 200 nm); increase in the CO_2 yield and simultaneous decrease in the CO yield	Not investigated	[7]
[1,2-phenylenebis(azanediyl)-bis(2-hydroxyl-5-diphenylphosphinylphenyl-methylene)] nickel (II)	Micro/nanorod	Self-assembly coordination reactions of diphenylphosphinyl groups, Schiff base (Salen-S), and nickel (II)	10, 15, 20, 25 wt.% (25 wt.%—optimal)	Diameter—500 nm; length—a few µm	Promotion of the formation of a char layer containing pyridine derivatives, phosphoric–oxygenic compounds, and nickel oxides blocking the heat and mass transfer; release of phosphorous-containing volatiles capturing small molecules, thus disrupting the combustion chain reactions	Decrease in the second degradation step temperature of 70 °C; increase in the residue yield at 700 °C; significantly improve LOI; decrease in the peak CO production; decrease in the smoke optical density	Slight decrease in tensile and impact strength	[8]
Titanium(IV) oxide TiO_2	Not specified	Not specified	6 wt.%	20 nm	Not investigated	Decrease in the pHRR and THR; significant decrease in FIGRA;	Not investigated	[9]

Nanoparticle	Form	Synthesis method	Content	Size	Mode of action	Effect on the thermal stability and flammability	Effect on the mechanical properties	Reference
						decrease in the TSR and maximum value of D_s		
Allylamine exfoliated alpha zirconium phosphate	Layered hexagon	Exfoliation of α-ZrP via the Brønsted acid-base interaction with allylamine	1, 2.5, 5, 7.5, 10 wt.% (10 wt.%—optimal)	4 nm	Heat barrier effect, blocking heat transfer into polymer matrix	Significantly improve LOI; increase in the main degradation step temperature of 100 °C; increase in the residue yield at 700 °C	Significant increase in the tensile and flexural strength	[10]
Cu_2O–TiO_2—graphene oxide	Nanosheet	Hydrothermal reaction with graphene oxide, tertbutyl titanate, and copper acetate	2 wt.%	Width—from 30 nm to 80 nm; thickness—several nanometers	The absorption of long-chain pyrolysis products of UPR onto acid sites of nanosheets, cracking into lower-carbon products and their catalytic carbonization by Cu_2O and TiO_2; thus, the formation of the char layer; nano-barrier effect of nanosheets extending the time of catalytic reaction; enhancement of the CO_2 production by the catalytic effect of Cu_2O on the CO molecules	Negligible effect on the thermal stability; decrease in the pHRR and THR	Not investigated	[11]
$Ti_3C_2T_x$ (MXene)	Nanosheet	Hydrofluoric acid etching of MAX (Ti_3AlC_2)	2 wt.%	Not specified	Promotion of the formation of the char layer inhibiting the heat and smoke transfer; the catalytic effect of TiO_2 inhibiting the release of smoke and toxic gaseous products	Decrease in the pHRR and THR; decrease in the TSP (decrease in the CO and CO_2 yields); increase in the char residue	Increase in the tensile strength; decrease in the elongation at break	[12]
MgAl DS LDH	Nanosheet	Intercalation of dodecyl sulfate by magnesium aluminum layered double hydroxide; co-precipitation and ion exchange	1, 2 wt.% of MgAl LDH (1 wt.%—optimal) in combination with 23 and 24 wt.% of TXP (24 wt.%—optimal)	Not specified	Dilution of the combustible gases by release of H_2O; release of metal oxides catalyzing char formation; synergistic effect with TXP	Increase in the thermal stability; increase in the residue yield at 600 °C	Not investigated	[13]
NiFe LDH coated with a coating containing P and Si	Nanosheet	Hydrothermal reaction of Ni^{2+}, Fe^{3+}, NH_4^+, and NO_3^-, and application of coating containing P and Si	Not specified	Average arithmetic roughness of fabric and LDH—367 nm	Release of pyrophosphoric acid from coating catalyzing char formation; char additionally stabilized by silicon compounds; release of interlayer water molecules diluting combustible gases, and interlayer metal ions, generating metal oxides	Increase in the thermal stability; increase in the residue yield at 800 °C; decrease in the HRR; decrease in the THR	Increase in the tensile strength	[14]

Nanoparticle	Form	Synthesis method	Content	Size	Mode of action	Effect on the thermal stability and flammability	Effect on the mechanical properties	Reference
					catalyzing char formation; formation of a char layer inhibiting heat and toxic gases emission			
POSS-functionalized GO	Not specified	Functionalization of GO on POSS-containing NH_2 groups through peptide bonds with COOH groups	0.05, 0.08, 0.1, 0.3 wt.% (0.1 wt.%—optimal)	Not specified	Barrier effect, blocking mass and heat transfer	Increase in thermal stability; increase in the residue yield at 800 °C; increase in LOI	Increase in the tensile strength	[15]
POSS-functionalized MMT	Not specified	Functionalization of MMT on POSS-containing NH_2 groups	0.5, 1, 3, 5 wt.% (3 wt.%—optimal)	Not specified	Barrier effect, blocking mass and heat transfer	Increase in thermal stability; increase in the residue yield at 800 °C	Increase in the tensile strength	[16]
OMPS	Not specified	Not investigated	1, 3, 5, 10 wt.% (10 wt.%—optimal)	Not specified	Barrier effect, blocking mass and heat transfer	Increase in thermal stability; increase in the residue yield at 800 °C	Not investigated	[17]
Nano-active modified pumice in a mixture of ATH, SS, and BA	Not specified	Sol-gel synthesis of silica gel through the synthesis of silica-rich pumice particles and sodium silicate	10 wt.% (1 wt.% of nAFPP—optimal)	30 nm	Dilution of the combustible gases by release of H_2O; promotion of the formation of the char layer	Decrease in the mass loss at the second degradation step; increase in the residue yield at 600 °C	Increase in the impact strength; decrease in the flexural strength	[18]
Boron nitride nanosheets containing phosphorus, nitrogen, and silicon	Nanosheet	High-temperature annealing and ultrasonic hydrolysis of boron nitride nanosheets, amination by the (3-aminopropyl)triethoxysilane, and the Michael addition reaction with hyperbranched polyphosphate acrylate	1, 3, 5 wt.% (3 wt.%—optimal)	Lateral dimensions—from 100 nm to 200 nm	Release of phosphorous-containing volatiles and electron-deficient boron-capturing free radicals, thus disrupting the combustion chain reactions; nano-barrier effect blocking the heat and mass transfer; dilution of combustible gases by release of NH_3; promotion of the formation of char layer by pyrolyzed phosphorus segments and its thermal stabilization by silicon segments	Decrease in the pHRR and THR; decrease in the CO and CO_2 yields; decrease in the temperature of the first stage of thermal degradation; decrease in the maximum mass loss rate	Not investigated	[19]

Table S2. Combustion characteristics of nano FRs with regard to pure UPR.

Group of nano FRs	Compound	Content	LOI	pHRR	Smoke production	Reference
Clay-based	Cloisite 25A	10 wt.%	Not investigated	• 705 kW/m^2 (reduced by 39%)	• TSR 818 m^2/m^2 (increased by 11%)	[20]

Group of nano FRs	Compound	Content	LOI	pHRR	Smoke production	Reference
	Cloisite25A (with APP)	5 wt.% (20 wt.% of APP)	Not investigated	• 417 kW/m^2 (reduced by 64%)	• TSR 796 m^2/m^2 (increased by 5%)	
	Organic-modified montmorillonite with methyl dihydroxyethyl hydrogenated tallow ammonium	3 wt.%	Not investigated	• 353 kW/m^2 (reduced by 31%)	• Not investigated	[1]
	Ionic-liquid-functionalized imogolite nanotubes (with APP)	0.4 wt.% (16.6 wt.% of APP)	• 28% (increased by 7.2%)	• 516.68 kW/m^2 (reduced by 22%)	• CO yield 0.080% (increased by 116%)<br>• CO_2 yield 1.074% (reduced by 31%)	[2]
Carbon-based	Multi-walled carbon nanotubes embedded nickel ferrite	2 wt.%	Not investigated	• 335 W/g (reduced by 69%)	Not investigated	[3]
	Multi-walled carbon nanotubes coated with g-C_3N_4 doped with boron and phosphorus	2 wt.% of BPCNTs, 18 wt.% of APP	• 30.6% (increased by 10.8%)	• 255.8 kW/m^2 (reduced by 61%)	• CO yield 0.21 kg/kg (increased by 9%)<br>• CO_2 yield 1.95 kg/kg (reduced by 63%)	[4]
	Pre-expanded graphite container for flame retardants	7 wt.%	• 33% (increased by 9.2%)	• 499 kW/m^2 (reduced by 9%)	• Peak of CO production yield 0.011 g/s (same as pure UPR)<br>• Peak of CO_2 production yield 0.328 g/s (reduced by 9%)	[5]
Nano-scale transition metal materials	Zinc(II) oxide	3 wt.%		Not investigated		[6]
	Cuprous(I) oxide	Not specified	• Not investigated	• 500 W/g (reduced by 17%)	• Peak of CO yield 1.5% (reduced by 17%)<br>• Peak of CO_2 yield 0.098% (increased by 5%)	[7]
	Nanorods containing nickel(II)	25 wt.%	• 38% (increased by 20%)	• 327 kW/m^2 (reduced by 69%)	• Peak of CO production yield 0.36 g/s (reduced by 66%)<br>• Peak of CO_2 production yield 0.31 g/s (reduced by 60%)	[8]
	Titanium(IV) oxide	6 wt.%	Not investigated	• 530.95 kW/m^2 (reduced by 35%)	• D_s max 638 [-] (reduced by 17%)	[9]

Group of nano FRs	Compound	Content	LOI	pHRR	Smoke production	Reference
	Allylamine exfoliated alpha zirconium phosphate	10 wt.%	• 27.1% (increased by 9.1%)	Not investigated	Not investigated	[10]
	Ti_3C_2Tx (MXene) nanosheets	2 wt.%	Not investigated	• 523.47 kW/m^2 (reduced by 30%)	• Peak of CO production yield 0.013 g/s (reduced by 32%)<br>• Peak of CO_2 production yield 0.357 g/s (reduced by 28%)	[12]
	Cu_2O–TiO_2–graphene oxide dual nanosheets	2 wt.%	Not investigated	• 631 kW/m^2 (reduced by 30%)	Not investigated	[11]
Layered Double Hydroxides	Dodecyl sulfate intercalated magnesium aluminum nitrate LDH	1 wt.%	Not investigated			[13]
	Nickel iron nitrate LDH	Not specified	Not investigated	• 487.89 kW/m^2 (reduced by 37%)	Not investigated	[14]
Polyhedral oligomeric silsesquioxanes	POSS-functionalized graphene oxide	1 wt.%	• 24.6% (increased by 2%)	Not investigated		[15]
	POSS-modified MMT	3 wt.%	Not investigated			[16]
	POSS-modified octamaleimide	10 wt.%	Not investigated			[17]
Other	Nano-active modified pumice	10 wt.% (1 wt.% of nAFPP)	Not investigated			[18]
	Boron nitride nanosheets	3 wt.%	Not investigated	• 870 kW/m^2 (reduced by 28.2%)	• Peak of CO yield 0.034 g/s (reduced by 21%)<br>• Peak of CO_2 yield 0.62 g/s (reduced by 35%)	[19]

Table S3. Environmental and health effects of nano flame retardants in unsaturated polyester resin.

Group of nano FRs	Environmental effects	Health effects
Clay-based	• Modification of clays accounts for nearly 50% of the energy consumption and emissions of acidifying substances and for ca. 30% of the global warming effects considering their production process [21]	• The cellular exposition may result in mitochondrion damage, a decrease in cell proliferation, the generation of reactive oxygen species, and damage the DNA<br>• Non-modified nanoclay and its thermally degraded product exhibit lower cellular toxicity compared to modified nanoclay [22]
Carbon-based	• Production of carbon nanofibres is the most energy-consuming step in the process of manufacturing UPR/CNF nanocomposites<br>• Energy consumption for manufacturing of carbon nanotubes and nanofibres is very high in comparison to other nano FRs<br>• Chemical modification of nanotubes requires a lower impact (from 40% to 80%) on the environment in comparison to physical processes [21]	• Safe when well-functionalized<br>• Non-modified exhibit high toxicity to human and animal cells<br>• Carbon nanomaterials may inhibit the proliferation of tumor cells [23]
Nano-scale transition metal materials	• Chemical manufacturing processes of TiO_2 require less energy and release lower amounts of greenhouse gases in comparison to physical and biological processes; however, physical methods are easier to apply at industrial scale<br>• Manufacturing of TiO_2 has a lower environmental impact in comparison to other nano FRs [21]	• Oxides may cause genotoxicity, cytotoxicity, and immunotoxicity<br>• Copper oxide may cause inflammation and penetrate further into the body, accumulate in the lungs, causing the generation of reactive oxygen species and oxidative stress; oral exposure can expose them to hepatotoxic effects and ulcer formation [24]<br>• Titanium dioxide is a suspected carcinogen; accumulates in digestive, respiratory, and reproductive system structures; may adversely affect the development of the ovum, and influence the offspring's health [25]
Layered Double Hydroxides (LDHs)	Not investigated	• Low toxicity [26]
Polyhedral oligomeric silsesquioxanes (POSS)	Not investigated	• Low toxicity<br>• Biocompatible [27]

Group of nano FRs	Environmental effects	Health effects
Nanocellulose	• Although the material has a natural origin, its environmental burden is high and comprises high water and fertilizer usage [28]	• May persist in the lungs, and its inhalation has a deleterious effect on human health<br>• No dermal and oral toxicity [29]

References

1. Nguyen, Q.T.; Ngo, T.D.; Bai, Y.; Tran, P. Experimental and numerical investigations on the thermal response of multilayer glass fibre/unsaturated polyester/organoclay composite. *Fire Mater.* **2016**, *40*, 1047-1069, doi:10.1002/fam.2364.
2. Zhu, T.; Guo, G.; Li, W.; Gao, M. Synergistic Flame Retardant Effect between Ionic Liquid-Functionalized Imogolite Nanotubes and Ammonium Polyphosphate in Unsaturated Polyester Resin. *ACS Omega* **2022**, *7*, 47601-47609, doi:10.1021/acsomega.2c02803.
3. Yu, X.; Wang, D.; Yuan, B.; Song, L.; Hu, Y. The effect of carbon nanotubes/$NiFe_2O^4$ on the thermal stability, combustion behavior and mechanical properties of unsaturated polyester resin. *RSC Adv.* **2016**, *6*, 96974–96983. https://doi.org/10.1039/C6RA15246E.
4. Chen, Z.; Zhang, W.; Yu, Y.; Chen, T.; Zhang, Q.; Li, C.; Jiang, J. Multi-walled carbon nanotubes encapsulated by graphitic carbon nitride with simultaneously co-doping of B and P and ammonium polyphosphate to improve flame retardancy of unsaturated polyester resins. *Mater. Chem. Phys.* **2022**, *277*, 125594, doi:10.1016/j.matchemphys.2021.125594.
5. Hu, W.-J.; Li, Y.-M.; Hu, S.-L.; Li, Y.-R.; Wang, D.-Y. The design of the nano-container to store the highly efficient flame retardants toward the enhancement of flame retardancy and smoke suppression for the unsaturated polyester resins. *Colloids Surf. A Physicochem. Eng. Asp.* **2023**, *658*, 130708. https://doi.org/10.1016/j.colsurfa.2022.130708.
6. Chen, H.; Tian, X.; Liu, J. Unsaturated Polyester Resin Nanocomposites Containing ZnO Modified with Oleic Acid Activated by N,N′-Carbonyldiimidazole. *Polymers* **2018**, *10*, 362, doi:10.3390/polym10040362
7. Hou, Y.; Hu, W.; Gui, Z.; Hu, Y. Effect of cuprous oxide with different sizes on thermal and combustion behaviors of unsaturated polyester resin. *J. Hazard. Mater.* **2017**, *334*, 39–48. https://doi.org/10.1016/j.jhazmat.2017.03.051.
8. Li, Z.; Fu, T.; Lu, J.-H.; He, J.-H.; Li, W.-D.; Liu, B.-W.; Chen, L.; Wang, Y.-Z. Ultra-high fire-safety unsaturated polyesters enabled by self-assembled micro/nano rod from Schiff base, diphenylphosphinyl group and nickel (II) metal. *Compos. B. Eng.* **2022**, *242*, 110032, doi:10.1016/j.compositesb.2022.110032.
9. Zatorski, W.; Sałasińska, K. Combustibility studies of unsaturated polyester resins modified by nanoparticles. *Polimery* **2016**, *61*, 815–823. https://doi.org/10.14314/polimery.2016.815.
10. Pichaimani, P.; Arumugam, H.; Gopalakrishnan, D.; Krishnasam, B.; Muthukaruppan, A. Partially Exfoliated α-ZrP Reinforced Unsaturated Polyester Nanocomposites by Simultaneous Co-polymerization and Brønsted Acid–Base Strategy. *J. Inorg. Organomet. Polym. Mat.* **2020**, *30*, 4095-4105, doi:10.1007/s10904-020-01558-x.
11. Wang, D.; Kan, Y.; Yu, X.; Liu, J.; Song, L.; Hu, Y. In situ loading ultra-small Cu_2O nanoparticles on 2D hierarchical TiO_2-graphene oxide dual-nanosheets: Towards reducing fire hazards of unsaturated polyester resin. *J. Hazard. Mater.* **2016**, *320*, 504–512. https://doi.org/10.1016/j.jhazmat.2016.08.066.
12. Hai, Y.; Jiang, S.; Zhou, C.; Sun, P.; Huang, Y.; Niu, S. Fire-safe unsaturated polyester resin nanocomposites based on MAX and MXene: A comparative investigation of their properties and mechanism of fire retardancy. *Dalton Trans.* **2020**, *49*, 5803–5814. https://doi.org/10.1039/D0DT00686F.
13. Kaul, P.; Joel Samson, A.; Enoch, I.; Selvakumar, P. Synergistic effect of LDH on thermal and flame retardant properties of unsaturated polyester nano-composite containing TXP. *Adv. Mater. Proc.* **2017**, *2*, 351–356. https://doi.org/10.5185/amp.2017/513.
14. Chu, F.; Hou, Y.; Liu, L.; Qiu, S.; Cai, W.; Xu, Z.; Song, L.; Hu, W. Hierarchical Structure: An effective Strategy to Enhance the Mechanical Performance and Fire Safety of Unsaturated Polyester Resin. *ACS Appl. Mater. Interfaces* **2019**, *11*, 29436–29447. https://doi.org/10.1021/acsami.9b08734.
15. Divakaran, N.; Kale, M.B.; Senthil, T.; Mubarak, S.; Dhamodharan, D.; Wu, L.; Wang, J. Novel Unsaturated Polyester Nanocomposites via Hybrid 3D POSS-Modified Graphene Oxide Reinforcement: Electro-Technical Application Perspective. *Nanomaterials* **2020**, *10*, E260. https://doi.org/10.3390/nano10020260.

16. Divakaran, N.; Kale, M.B.; Dhamodharan, D.; Mubarak, S.; Wu, L.; Wang, J. Effect of POSS-Modified Montmorillonite on Thermal, Mechanical, and Electrical Properties of Unsaturated Polyester Nanocomposites. *Polymers* **2020**, *12*, 2031. https://doi.org/10.3390/polym12092031.
17. Jothibasu, S.; Chandramohan, A.; Kumar, A.A.; Alagar, M. Polyhedral oligomeric silsesquioxane (POSS) reinforced-unsaturated polyester hybrid nanocomposites: Thermal, thermomechanical and morphological properties. *J. Macromol. Sci. A.* **2018**, *55*, 433-439, doi:10.1080/10601325.2018.1453264.
18. Rakhman, A.; Diharjo, K.; Raharjo, W.W.; Suryanti, V.; Kaleg, S. Improvement of Fire Resistance and Mechanical Properties of Glass Fiber Reinforced Plastic (GFRP) Composite Prepared from Combination of Active Nano Filler of Modified Pumice and Commercial Active Fillers. *Polymers* **2023**, *15*, 14, doi:10.3390/polym15010051.
19. Wang, D.; Mu, X.; Cai, W.; Song, L.; Ma, C.; Hu, Y. Constructing phosphorus, nitrogen, silicon-co-contained boron nitride nanosheets to reinforce flame retardant properties of unsaturated polyester resin. *Compos. Part A Appl. Sci. Manuf.* **2018**, *109*, 546–554. https://doi.org/10.1016/j.compositesa.2018.04.003.
20. Nazaré, S.; Kandola, B.K.; Horrocks, A.R. Flame-retardant unsaturated polyester resin incorporating nanoclays. *Polym. Adv. Technol.* **2006**, *17*, 294-303, doi:10.1002/pat.687.
21. Carroccio, S.C.; Scarfato, P.; Bruno, E.; Aprea, P.; Dintcheva, N.T.; Filippone, G. Impact of nanoparticles on the environmental sustainability of polymer nanocomposites based on bioplastics or recycled plastics – A review of life-cycle assessment studies. *J. Clean. Prod.* **2022**, *335*, 130322, doi:10.1016/j.jclepro.2021.130322.
22. Wagner, A.; Eldawud, R.; White, A.; Agarwal, S.; Stueckle, T.A.; Sierros, K.A.; Rojanasakul, Y.; Gupta, R.K.; Dinu, C.Z. Toxicity evaluations of nanoclays and thermally degraded byproducts through spectroscopical and microscopical approaches. *Biochim. Biophys. Acta Gen. Subj.* **2017**, *1861*, 3406-3415, doi:10.1016/j.bbagen.2016.09.003.
23. Hassan, A.A.; Mansour, M.K.; Sayed El Ahl, R.M.H.; El Hamaky, A.M.A.; Oraby, N.H. Toxic and beneficial effects of carbon nanomaterials on human and animal health. In *Carbon Nanomaterials for Agri-Food and Environmental Applications*, Abd-Elsalam, K.A., Ed.; Elsevier: 2020; pp. 535-555.
24. Naz, S.; Gul, A.; Zia, M. Toxicity of copper oxide nanoparticles: a review study. *IET Nanobiotechnol.* **2020**, *14*, 1-13, doi:10.1049/iet-nbt.2019.0176.
25. Minghui, F.; Ran, S.; Yuxue, J.; Minjia, S. Toxic effects of titanium dioxide nanoparticles on reproduction in mammals. *Front. Bioeng. Biotechnol.* **2023**, *11*, 1183592, doi:10.3389/fbioe.2023.1183592.
26. Kura, A.U.; Ain, N.M.; Hussein, M.Z.; Fakurazi, S.; Hussein-Al-Ali, S.H. Toxicity and metabolism of layered double hydroxide intercalated with levodopa in a Parkinson's disease model. *Int. J. Mol. Sci.* **2014**, *15*, 5916-5927, doi:10.3390/ijms15045916.
27. Liu, S.; Guo, R.; Li, C.; Lu, C.; Yang, G.; Wang, F.; Nie, J.; Ma, C.; Gao, M. POSS hybrid hydrogels: A brief review of synthesis, properties and applications. *Eur. Polym. J.* **2021**, *143*, 21, doi:10.1016/j.eurpolymj.2020.110180.
28. Penloglou, G.; Basna, A.; Pavlou, A.; Kiparissides, C. Techno-Economic Considerations on Nanocellulose's Future Progress: A Short Review. *Processes* **2023**, *11*, doi:10.3390/pr11082312.
29. Stoudmann, N.; Schmutz, M.; Hirsch, C.; Nowack, B.; Som, C. Human hazard potential of nanocellulose: quantitative insights from the literature. *Nanotoxicology* **2020**, *14*, 1241-1257, doi:10.1080/17435390.2020.1814440.
